# The impact of unplanned school closure on children’s social contact: rapid evidence review

**DOI:** 10.2807/1560-7917.ES.2020.25.13.2000188

**Published:** 2020-04-02

**Authors:** Samantha K Brooks, Louise E Smith, Rebecca K Webster, Dale Weston, Lisa Woodland, Ian Hall, G James Rubin

**Affiliations:** 1 Department of Psychological Medicine, King’s College London, Weston Education Centre, London, United Kingdom; 2 Behavioural Science Team, Emergency Response Department Science and Technology, Public Health England, Porton Down, United Kingdom; 3 Department of Mathematics and School of Health Sciences, University of Manchester, Manchester, United Kingdom

**Keywords:** Infectious disease outbreaks, children, school, school closures, social contact

## Abstract

**Background:**

Emergency school closures are often used as public health interventions during infectious disease outbreaks to minimise the spread of infection. However, if children continue mixing with others outside the home during closures, the effect of these measures may be limited.

**Aim:**

This review aimed to summarise existing literature on children’s activities and contacts made outside the home during unplanned school closures.

**Methods:**

In February 2020, we searched four databases, MEDLINE, PsycInfo, Embase and Web of Science, from inception to 5 February 2020 for papers published in English or Italian in peer-reviewed journals reporting on primary research exploring children’s social activities during unplanned school closures. Main findings were extracted.

**Results:**

A total of 3,343 citations were screened and 19 included in the review. Activities and social contacts appeared to decrease during closures, but contact remained common. All studies reported children leaving the home or being cared for by non-household members. There was some evidence that older child age (two studies) and parental disagreement (two studies) with closure were predictive of children leaving the home, and mixed evidence regarding the relationship between infection status and such. Parental agreement with closure was generally high, but some disagreed because of perceived low risk of infection and issues regarding childcare and financial impact.

**Conclusion:**

Evidence suggests that many children continue to leave home and mix with others during school closures despite public health recommendations to avoid social contact. This review of behaviour during unplanned school closures could be used to improve infectious disease modelling.

## Introduction

Gaining control of an infectious disease outbreak can require making difficult decisions, particularly when infections are human-to-human transmissible. Children are often in close physical proximity at school, have less-than-perfect hygiene behaviours and have low prior immunity to many infections [1]. For this reason, school closures are often proposed as one way of delaying the spread of infection [2]. There is evidence to suggest that social contacts should reduce when schools are closed. For example, it has been reported that students have contact with fewer people during weekends [3] and that the number of contacts children have with others approximately halves during the holidays [4,5]. Several studies have also examined illness transmission rates during planned school closures, reporting a reduction in illness during school holidays [6-8] and teacher strikes [9].

However, school closure is not a step that can be taken lightly. Clearly, closures can have an impact on the education of the children involved. But they can also have an impact on the healthcare system, on the wider economy if large numbers of the workforce stay home to look after their children, on household incomes, on social policies implemented at school and on the likelihood of children engaging in other risky behaviours if they must be left unattended at home [10*]. Indeed, the secondary economic and social effects of school closures are potentially very large [11].

Understanding whether the effectiveness of school closure in terms of reducing the spread of disease outweighs these impacts is therefore important. One of the key unknowns is what happens to children after a school is closed. The optimum answer from an epidemiological perspective is that children remain in their homes for the duration of the closure, never coming into contact with another person [12,13]. However, this is impractical and from front-line experience of outbreak management, there are many accounts of children continuing to congregate after being sent home from school and sometimes engaging in behaviour likely to increase the risks of infection spreading [14,15]. Any full assessment of the impact of school closures should take this into account.

A related issue is the extent to which children have contact with people, particularly those in vulnerable groups, with whom they would not usually have contact on a typical school day following a school closure. While their number of social contacts may be lower following closures, children may, for example, be taken care of by grandparents which increases the likelihood of older adults who may be at risk coming into contact with the infectious disease in question.

Finally, given that school closures are often accompanied by advice to parents to limit the contact their children have with others, understanding what practical or attitudinal factors affect the likelihood of children mixing during a closure may also be helpful in improving the advice that is given out.

Given these considerations surrounding school closures, we aimed to summarise existing literature on children’s activities and contacts made outside the home during unplanned school closures in this rapid evidence review. To expand, we examined: (i) what is currently known about the impact of unplanned school closure on children’s interaction with others outside the home, (ii) who provides childcare during a closure, (iii) what factors are associated with children interacting with others outside the home during a closure, and (iv) what affected parents think about closures.

## Method

This work was carried out as a rapid evidence review in response to the COVID-19 outbreak that began at the end of 2019, and which has led to policymakers across the world discussing how best to minimise the spread of the disease. Rapid reviews follow the general principles of a systematic review but may be simplified, for example, by not including grey literature or conducting a full quality appraisal, in order to produce information in a shorter period of time with minimal impact on quality. They are essential in circumstances such as the developing situation with COVID-19 as policymakers urgently need synthesised evidence in order to make informed decisions regarding guidelines for the public. As there are no specific guidelines and no standardised methodology for rapid reviews, the PRISMA checklist has not been completed. However, the only Preferred Reporting Items for Systematic Reviews and Meta-Analyses (PRISMA) [16] checklist items that this study lacks relate to the analysis of risk of bias in individual studies; because of time constraints, a quality assessment of each paper was not conducted.

### Search strategy and selection criteria

We used the following search strategy to search abstracts and titles in MEDLINE, PsycInfo and Embase:

1. school* ADJ3 close* OR ADJ3 closure* OR ADJ3 closing* OR ADJ3 dismiss*
2. nurser* ADJ3 close* OR ADJ3 closure* OR ADJ3 closing* OR ADJ3 dismiss*
3. kindergar* ADJ3 close* OR ADJ3 closure* OR ADJ3 closing* OR ADJ3 dismiss*
4. playgroup* ADJ3 close* OR ADJ3 closure* OR ADJ3 closing* OR ADJ3 dismiss*
5. play-group* ADJ3 close* OR ADJ3 closure* OR ADJ3 closing* OR ADJ3 dismiss*
6. 1 OR 2 OR 3 OR 4 OR 5
7. behaviour* OR behaviour* OR contact* OR mix* OR social* OR targeted layered containment
8. 6 AND 7

We repeated the same search on Web of Science using NEAR instead of ADJ3. All databases were searched from inception to 5 February 2020.

### Inclusion and exclusion criteria

To be included in the review, studies had to: (i) report on primary research, (ii) be published in peer-reviewed journals, (iii) be written in English or Italian, the languages spoken by our team, and (iv) report on social activities of children during unplanned temporary school closures because we speculated that mixing behaviour will likely be different during closures with plenty of notice, giving parents more time to plan what to do.

We excluded papers based on intentions, hypothetical scenarios or simulations.

### Screening

One author, SKB, ran the search strategy on all databases and downloaded all resulting citations to EndNote version X9 (Thomson Reuters, New York, United States (US)). Titles and then abstracts were all screened for relevance according to the inclusion criteria by at least two authors (SKB, LES, RKW, DW or LW). The authors compared which texts they had chosen for inclusion and discrepancies were resolved through discussion with the wider team. Full texts of all remaining citations were obtained and reviewed by one author (SKB), excluding any that did not meet all inclusion criteria. Finally, the reference lists of remaining papers were hand-searched by SKB for any additional relevant studies. A flowchart of the screening process is presented in the Figure.

**Figure:**
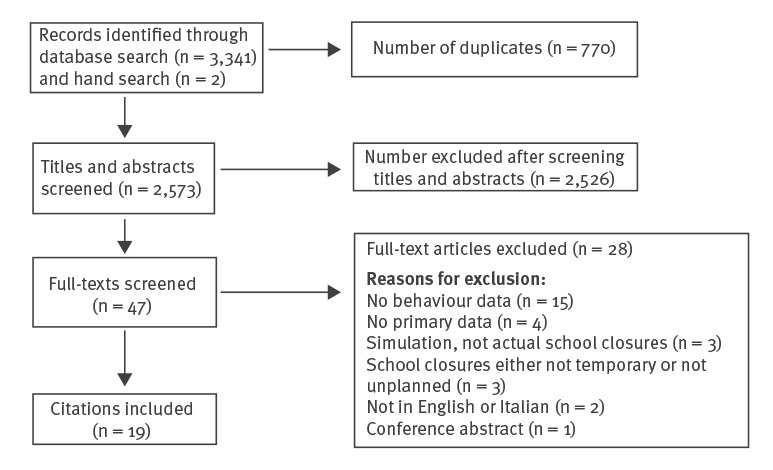
Flowchart of the screening process for the rapid evidence review of the impact of unplanned school closure on children’s social contact, February 2020

### Data extraction

We designed spreadsheets to extract the following data from papers: authors, publication year, country of study, design, participants (including number and demographic information), reason for school closure, length of school closure and key results (i.e. behaviours during school closures, number of children leaving the home during closures and number of children who were cared for by non-household members). With regards to childcare arrangements, we were only interested in arrangements that involved a non-household member, e.g. grandparent, family friend or babysitter, rather than household members, e.g. a parent working from home or an older sibling, in order to explore how many children had contact with people they would not already have contact with by living in the same home. We were also interested in the number of children left home alone. Data extraction was carried out by one author (SKB).

## Results

Database searches yielded 3,341 papers and two additional papers were identified via hand-searching; 770 duplicates were removed and the remaining 2,573 were screened for relevance. After this screening, a total of 19 papers remained and were included in the review, 18 of which [17-34] used a cross-sectional design employing questionnaires to assess difficulties during the school closures, activities outside the home during the closures and/or who provided childcare during the closures. The remaining paper [14] used a qualitative design. The majority (n = 10) were from the US [19,21,23-25,27,30,32-34]; four papers were from Australia [14,20,22,29] and the remaining papers were from Argentina [28], Japan [26], Russia [18], Taiwan [31] and the UK [17]. Most papers reported on school closures because of the 2009 influenza A(H1N1) pandemic (n = 12) [14,17,19,20,22,24-26,28,29,31,32] or other influenza or influenza-like outbreaks (n = 6) [18,21,23,27,30,33]. One paper reported on a school closed in preparation for a hurricane [34]. The duration of school closures ranged from 1 day [32] to 2 weeks [28]. The size of the quantitative studies ranged from 35 households (representing 67 children) [21] to 2,229 households (representing 4,171 children) [34]. The Table provides a summary of activities outside the home and childcare arrangements involving non-household members during school closures. See Supplement S1 for more detail on the results of each study.

**Table:** Studies included in the rapid review and summary of findings about activities outside the home and childcare arrangements involving non-household members during school closures (n = 19)

Study, year and place	Participants	Activities outside the home	Childcare arrangements involving non-household members
Basurto-Davila et al. (2013), Argentina [28]	226 households; children aged 6–15 years from three schools closed for 2 weeks because of influenza A(H1N1).	67% of children visited public places at least once; 45% left the home several times.	Left with a relative or family friend (82%/88% depending on region), hired nanny (13%/5%), other special arrangements (3%/4%), left alone (2%/1%).
Braunack-Mayer et al. (2013), Australia [14]	Four school principals, 25 staff, 14 parents, 13 students aged 12–17 years; schools either partially or fully closed because of H1N1 (length of closure unclear).	Qualitative study indicating most people adhered to advised quarantine, but in the absence of clear instructions, many invented their own rules. Some parents quarantined their children to avoid being seen as irresponsible. However, many parents reported their children were home alone and so it was unclear whether they complied. Others reported seeing the closure as ineffective and did not quarantine their children. One student reported meeting friends regularly even though his parents believed he was at home.	Not reported.
Effler et al. (2010), Australia [20]	233 households; median age of children 11 years (range: 5–13); three schools closed because of H1N1; School A closed entirely ‘for the coming week’ while Schools B and C cancelled classes for grades 5 and 5–7, respectively.	74% participated in activities outside the home on at least one occasion, reporting a total of 860 out-of-home activities with an average of 3.7 out-of-home activities per student.	Asymptomatic students: with children other than their siblings (19%). Ill students: with children other than their siblings (6%). All students: left alone for at least some time (10%).
McVernon et al. (2011), Australia [29]	314 households; 33 schools; schools with confirmed cases of H1N1 in multiple classes were entirely closed for 7 days while schools with confirmed cases in only one class were instructed to close only that class.	43 households reported that a child spent at least 1 day outside the family home and mixing with other children occurred on almost half of these occasions (48.8%). Contact with children who were not immediate family members was less likely during days spent at home. No child visited a household in which another child was ill, compared with reported child visitors in 15.9% of 226 homes without a case.	Households with influenza: adult from outside the home (44.4% for households that complied with advice to remain in home vs 2.4% for non-compliant households). Households without influenza: adult from outside the home (28.3% for households that complied vs 4.0% for non-compliant households).
van Gemert et al. (2018), Australia [22]	99 students with laboratory confirmed H1N1; age 6–17 years; Seven schools closed for 3–9 days (not including weekends).	26% (21/81) who reported usually taking part in extra-curricular activities (not sports or religious activities) continued to take part in extra-curricular activities.	Not reported.
Mizumoto et al. (2013), Japan [26]	882 households; 25.2% in kindergarten, 24.8% in primary school, 25.1% in junior high school and 24.9% in high school; age range 4–18 years; ‘school closure or class suspension at least once’ because of H1N1.	20.5% left the home for non-essential reasons.	Another household member (64.3%), left alone (28.5%), special arrangement such as parental absence from work (7.3%).
Litvinova et al. (2019), Russia [18]	450 participants including students and their household members; School A for children aged 6–17 years and School B for children aged 6–15 years; schools closed for 7 days to mitigate spread of seasonal influenza.	There was a reduction in the number of contacts made by students (14.2 contacts/day when open vs 6.5 when closed). Students reduced their number of contacts with individuals under 18 years of age (75% reduction) and 19–59-year-olds (20% reduction), while increasing contacts with individuals aged 60 years and over (52% increase), although the absolute value remained low (less than one contact/day).	Not reported.
Chen et al. (2011), Taiwan [31]	232 households; school for children aged 5–12 years; school closed for 7 days because of H1N1.	13% went to public places or gatherings at least once, 12% visited relatives, 5% went to parents' workplace.	Parents (60%), other relatives (35%), others (4%), left alone (1%).
Jackson et al. (2011), UK [17]	107 students (only 46 reported how many times they visited public places during closures); children aged 11–15 years; school closed for 1 week, reopened for 2 days, then closed for another week because of H1N1.	98% visited more than one place. 73 students provided their typical number of contacts per day during closure and 35 also provided information for a typical school day. Mean totals of reported contacts were 70.3 and 24.8 during typical school days and closure respectively.	Among caregivers for whom information was available, 125/182 (69%) would have seen the student on a typical school day.
Borse et al. (2011), US [25]	554 households; median age of children: 8 years; schools closed for 5–7 days because of H1N1.	30% of students visited at least one locale outside their homes.	Not reported.
Epson et al. (2015), US [21]	35 households, representing 67 students; one elementary school and one junior and senior high school housed in the same building complex; schools closed between 29 January 2013 and 5 February 2013 because of influenza-like illness.	58% visited at least one outside venue.	Adult from outside the household (9%), work with parents (6%), childcare programme (3%), left alone (9%).
Gift et al. (2010), US [24]	214 households, with 269 children under 18 years of age; elementary school closed for 1 week because of H1N1.	69% visited at least one other location.	Home as main location (77%). The next most common locations were another family member's home, non-family member's home, parents’ workplace, vacation, daycare and ‘other’.
Johnson et al. (2008), US [23]	220 households, with 355 children; median age of children: 12 years (range: 5–19); schools closed for 12 days because of influenza virus B.	89% visited at least one public location and 47% travelled outside of the county.	Special childcare arrangements including grandparents, other relatives, other adults, taking the child to work, having older siblings watch them or using childcare programs (10%), one or more night spent outside the household (3%).
Miller et al. (2010), US [19]	63 parents of 176 lower school students (grades 5–8); 188 upper school students (grades 9–12); week-long closure because of H1N1.	Upper school: Mean number of days spent on activities: 3.42 any other outdoor activity; 2.44 eating at restaurants; 1.89 using public transport; 1.48 hosting a friend; 1.47 shopping; 1.47 any other indoor activity; 0.44 working at a job. Average number of friends seen per day: 2.53 on Wednesday, 2.06 Thursday, 2.59 Friday, 2.40 Saturday, 1.23 Sunday, 1.02 Monday, 1.05 Tuesday. Lower school: Mean number of days spent on activities: 2.77 any other outdoor activity; 1.34 eating at restaurants; 1.12 any other indoor activity; 1.05 shopping; 0.73 visiting a friend; 0.55 hosting a friend; 0.10 using public transport. Average number of friends seen per day: 0.30 Wednesday, 0.52 Thursday, 0.84 Friday, 0.83 Saturday, 1.17 Sunday, 0.74 Monday, 0.68 Tuesday.	Upper school: Proportion of caregivers: 0.62 parent, 0.24 sibling, 0.07 grandparent, 0.07 other, 0.06 nanny/babysitter, 0.07 friend's caretaker, 0.11 other, 0.88 self. Lower school: Proportion of caregivers: 0.85 parent, 0.30 sibling, 0.09 grandparent, 0.15 other family, 0.27 nanny/babysitter, 0.03 friend's caretaker, 0.06 other, 0.76 self.
Russell et al. (2016), US [27]	99 households, representing 197 children; students in pre-kindergarten up to grade 12; school closed for 4 days because of influenza-like illness.	77% of children went outside the home or visited a non-household member, participating in a mean of two activities (IQR: 1–4).	Adult from outside the household (20%); childcare programme (1%).
Steelfisher et al. (2010), US [32]	523 parents; ages and number of children not reported; childcare centres and schools closed because of H1N1: 10% were closed for 1 day, 19% for 2 days, 29% for 3 days, 15% for 4 days, 17% for 5 days, 9% for more than 5 and 2% didn't know.	56% reported their child participated in at least one activity involving people outside the household.	81% were cared for by an adult in the household, 20% by a family member outside the household, 1% by a friend/neighbour, 3% by a professional care provider, and 10% stayed home alone.
Timperio et al. (2009), US [30]	262 households, representing 480 children; ages not reported. Two schools closed because of seasonal influenza; one closed for 3 days and the other for 4 days.	43.3% visited strip malls or WalMart, the largest store in the area; 42.9% visited family; 38.7% went grocery shopping; 32.6% ate at restaurants; 30.3% either visited friends’ homes or had friends visiting their home; 29.1% attended religious services; 23.8% took part in sports activities; 17.6% went to public gatherings such as concerts, movies or festivals; 8.4% went to a part time job.	Not reported.
Tsai et al. (2017), US [33]	208 households with 423 children; school closed for 8 days because of influenza.	Not reported.	Childcare programme (3%), attending work with parents (1%), left alone without supervision (1%), old enough to care for themselves (15%).
Zheteyeva et al. (2017), US [34]	2,229 households with 4,247 students; kindergarten to grade 12; schools closed for 4 days in preparation for Hurricane Isaac.	Not reported.	Old enough to care for themselves (11.6%), went to work with parents (5.3%), childcare programme (2.6%), left alone without supervision (2.5%).

IQR: interquartile range; UK: United Kingdom; US: United States.

### Interaction with others outside the home

Participation in activities and interactions with others did appear to decrease during school closures compared with regular school days [17-19]. For example, one study of 107 students aged 11 to 15 years in the UK [17] reported that school closure was associated with a 65% reduction in the mean total number of contacts for each student. However, social contact was still common: all 19 studies showed that at least some children took part in activities outside of the home during school closures, even despite health recommendations to remain indoors and isolated from others. In fact, eight studies [17,20,21,23,24,27,28,32] showed that the majority of children (i.e. more than 50%) left the home or took part in activities involving non-household members, including the UK study of school closures during the H1N1 outbreak which found that 98% of children left their homes during that time [17].

### Factors associated with contact outside the home

#### Infection status

Several studies suggested that children who reported illness during a school closure were less likely to take part in activities outside the home [17,20-22]. For example, in a study of 233 Australian households (children with a median age of 11 years), Effler et al. [20] reported a statistically significant difference for the proportion of cases, i.e. students testing positive for influenza A(H1N1) virus, students who had been in close physical proximity to cases, and peers who did not meet case or contact criteria who reported leaving the home more than once during the closure period (42%, 66% and 92%, respectively) (p < 0.0001). Cases reported an average of 0.8 out-of-home activities per student per week, compared with 2.9 for contacts and 5.6 for peers. Other studies reported that children who reported illness or lived in households in which influenza-like illness was reported did not participate in the majority of activities reported by other students [21,22] and that their contact with others was reduced [17].

However, other studies reported few differences in out-of-home activities between symptomatic and asymptomatic children [19,23-26]. For example, one American study of 176 children in grades 5 to 12 [19] found that students with illnesses were more likely to report an increase in travel plans; the reasons for this are not clear. Two other American studies found that children with an influenza-like illness were more likely to have visited a healthcare provider ((p<0.01) [24], statistics not reported [25]) but no other differences in out-of-home activities were found between students with and without symptoms [24,25].

#### Age

Three American studies noted more activities and contacts among older children [19,23,27]. In the study by Miller et al. [19], grade 12 students, i.e. students aged 16 to 18 years, had more contacts than students in other grades during closures, particularly late in the week. The authors suggest that because many grade 12 students were not regularly attending classes at the school before the outbreak, they may have felt that they or their friends had not been exposed to the infection. One study of 355 children [23] found that children 12 years of age and over were significantly more likely to go to fast food restaurants and parties (p < 0.05), but less likely to go grocery shopping than children 12 years of age and under.

Conversely, one Japanese study of 882 households, with children of kindergarten to high school age [26], found that younger children were more likely to leave the home during a closure; 53.2% of kindergarten pupils, 42.5% of primary school pupils, 30.3% of junior high school pupils and 33.2% of high school pupils reporting that they left the home at least once. Primary school pupils were significantly more likely to leave the home to visit a supermarket or convenience store (p = 0.05 for the association between school category and shopping), while junior high school pupils and primary school pupils were significantly more likely to leave the home to attend extracurricular studies compared with pupils in other school categories (p = 0.02).

#### District

Evidence from one study of behaviour in children aged 6 to 15 years from 226 households in two different school districts in Argentina [28] suggested that location can affect the out-of-home activities children take part in during school closures. In this study, children in Jujuy were more likely to attend religious events, use public transport, and go to plazas and recreation areas than children in Ushuaia. Meanwhile, children in Ushuaia were more likely to go to the movie theatre and restaurants than children in Jujuy. The study suggested socioeconomic differences may well be the reason for this: Ushuaia has one of the lowest poverty rates in the country, whereas Jujuy has one of the highest.

#### Employment status of adults in the household

A study of 554 households in the US (median age of children: 8 years) found that if all adults in the home were employed, ill children were less likely to leave the home [25]. The probability of a child visiting any other venue was 34% if the child came from a household where at least one adult was not employed, with annual income less than USD 25,000 and with only one child between kindergarten and fourth grade age who did not have an influenza-like illness before or during the closure. However, if all adults in the household were employed, the probability of children leaving the home decreased to 24%. This was an unexpected finding as we would have expected that children living only with employed adults might have to leave the home for childcare arrangements. The authors did not offer reasons for the association between employed adults and reduced likelihood of children leaving the home.

#### Perceived appropriateness of school closure

Two studies, one from Australia and one from Japan, found that parental opinion about the appropriateness of the school closure was significantly correlated with student participation in activities outside the home (p = 0.0006 and p = 0.03 respectively) [20,26]. Students of parents who thought the school closure was not appropriate reported a mean of 4.7 out-of-home activities during the closure, compared with a mean of 4.3 activities for students of parents who were unsure and 2.8 for students of parents who thought the closure was appropriate [20]. This pattern persisted when the analysis was restricted to the 202 students who were asymptomatic. Similarly, Mizumoto et al. [26] found that proportionately fewer children left the home in households that believed the closure was appropriate: 38.8% compared with 53.2% of children in households who felt the closure was inappropriate.

#### Extent of closure

One Japanese study of 882 households [26] found that extent of school closure was significantly associated with the frequency of children leaving the home: closure of the entire school, closure of a single grade or single class suspension were associated with 47.8%, 32.2% and 40.3% of children leaving the home, respectively (p = 0.01).

#### Length of time advised to isolate

One Australian study of 314 households investigated adherence with reactive school closure attempting to contain the H1N1 pandemic [29]. Participants had been asked to go into voluntary home quarantine ranging from 1 to 14 days in length. Children stayed at home for more than 94% of the days they were advised to be in quarantine. This figure was not associated with the length of quarantine nor did it fluctuate over the course of the quarantine period.

#### Day of the week

In one American study [19], contact rates of uninfected students at the end of the week were lower than at the beginning. Based on visual inspection of the graph presented in the study, contacts substantially increased for older children, i.e. children in grades 11 and 12, on Friday and Saturday.

#### Special childcare arrangements

A study of 882 households in Japan found that children in households where special childcare arrangements were needed during closure were significantly more likely to leave the home than households in which children were independent and able to take care of themselves (53.1% vs 35.9%; p < 0.01) [26].

#### Other factors considered

Based on a study of 882 Japanese households, a child’s sex, household educational level, household income and household size were not associated with the likelihood of the child leaving the home during school closure [26].

### Parental attitudes towards school closure

#### Perceived benefit of closure

Parents generally agreed with school closures. Several studies reported high rates of parents being at least moderately supportive of the closure: 97% [30], 93% [26], 91% [23], 78% [28], 73% [31] and 71% [32]. The main reasons for agreeing with school closures were believing that it would protect the health of the community, the children themselves and the household. Another main reason was believing that there were too many sick children for the school to remain open [20,23,26,30]. Timperio et al. [30] found that over 90% of parents from 262 households in the US felt it was important to disinfect the schools while closed to reduce the community spread of influenza.

#### Perceived risk of infection

Several of the main reasons for disagreeing with school closures appeared to be related to perceived risk: parents cited beliefs that closures do not protect against influenza [28], that the illness is only mild [20,26] and that school closure is not an effective measure against infection [26].

#### Practicalities of school closure

Other main reasons for disagreeing with school closures were related to the practicalities and subsequent impact of the closure. For example, parents were concerned about the impact on the child’s education [28], difficulties making childcare arrangements [26] and concerns about the economic impact [20,23]. Parents reported various difficulties associated with school closures, primarily lost income, the effort of arranging alternate childcare and uncertainty about the duration of the closure [33,34]. Some studies also illustrated a lack of consistency by schools regarding the importance of not participating in social activities. For example, 17% of parents reported that after-school activities were not cancelled [32] while others noted that school athletic events were still held on days that school was closed [30].

## Discussion

This review of 19 papers found that all studies reported children leaving the home during the closure period and/or being looked after by non-household members, thereby having social contact with others they could potentially infect if they themselves were infected. There was some evidence that continuing to engage in social contact during school closures may be related to older child age, parental disagreement with closure and potentially infection status.

During a major infectious disease outbreak, school closure has the potential to slow the spread of infection. However, the effects of a closure will be attenuated if children continue to mix. Of the 19 papers that we identified, all reported that some degree of mixing continued to occur outside of the home. We should not be surprised at this. Even for adults, self-isolation can be difficult [35] and stressful [36], and children often have wider social circles and feel more social pressure to interact. The precise extent to which contact patterns change during a closure is harder to determine. Only a limited number of studies have attempted to quantify this, reporting reductions in the number of contacts from 70.3 on typical school days to 24.8 [17] and 14.2 to 6.5 [18] during closures. The difference in rates reported are likely because of social and cultural differences as well as differences in definitions of a ‘contact’ between the papers: there appear to be various definitions of ‘social contacts’ in addition to what vicinity and duration are necessary for an encounter to be considered a ‘contact’.

Complicating matters is that the qualitative nature of contacts also changes. The studies included in this review explored what types of activities children engage in outside of the home during a closure (Table). These include a large range of recreational and social activities, from shopping to meeting friends indoors, using public transport and visiting restaurants. It is likely that the type of activity is important in determining the likelihood of infection spreading. For example, participation in sports events have been noted to be particularly associated with the spread of influenza, as have social events such as parties, whereas visits to a park or beach are reported as being less likely to result in disease spread [20].

We conclude that further research is needed to quantify the rates of contact associated with the various activities reported in this review; contacts in households, schools and workplaces are likely of more sustained duration than contacts in more transient social settings such as shopping. However, social gatherings such as parties may form a ‘middle ground’ in that they likely involve less sustained contact than in a household or school, but more than in a grocery store for example, and the acceptability of such social gatherings is likely to differ across the population. Assuming infection given a contact is a function of duration and type of contact, this can form the basis of more evidence-based modelling and risk assessment.

Reassuringly, our review found that relatively few children required special childcare arrangements that might actively increase the risk of disease transmission, such as being placed into a semi-formal childcare arrangement with other children or being looked after by grandparents. While low, the proportion of children left home alone unsupervised, however, is of concern because unsupervised children could potentially leave the home without their parents knowing thus risking infection spread. If school closures are considered in the future, public health officials should consider how best to support parents and prevent this from occurring.

We found unclear evidence about the majority of the other predictors of out-of-home activities. In particular, there was mixed evidence about whether children showing symptoms of illness or who have been ill during the closure will take part in similar out-of-home activities compared with children who are not ill. We find it particularly concerning that even symptomatic children are participating in out-of-home activities.

Different studies found that both older age and younger age were associated with leaving the home during school closures. It may be that the direction of findings depends on the activity in question. For example, younger children seem to be more likely to go grocery shopping, perhaps because they are too young to be left at home alone when their caregiver goes to the shops, whereas older children are more likely to take part in social activities like parties and going to restaurants. It should be noted that the one study showing younger children were more likely to leave the home [26] was the only study from Japan so the difference in findings may relate to cultural differences.

Parental attitudes associated with agreeing or disagreeing with school closures were similar to those seen in relation to other preventive health behaviours for infectious diseases [37,38]. In particular, two of the studies included in this review suggested there was a strong association between allowing children to socialise outside the home and disagreeing with the school closure [20,26]. Ensuring parents understand why school closure is important will be a key factor determining the success of the measure in any future disease outbreak. In this regard, it was concerning that two studies appeared to highlight a lack of clarity in terms of advice about whether children could take part in social activities and knowing what children were and were not advised to do [30,32]. Advice from schools should be consistent with public health advice; hosting extra-curricular activities and sporting events during a closure sends mixed messages to parents and can be confusing or detrimental [14].

In terms of how our findings fit with the wider literature, one particular discrepancy is worth noting. Evidence from studies in which people are asked how they would react to a hypothetical school closure often find that parents believe they would co-operate with public health advice. For example, one study involving a hypothetical scenario of schools closing for 3 months because of an influenza pandemic found that 85% of parents responsible for children aged between 5 and 17 years of age believed they would be able to keep their children from taking public transport, going to public events and gathering outside the home during this lengthy school closure period [39]. Meanwhile, another found that 96.7% of parents claimed they would keep their children away from others for a month if schools and child-care facilities were closed [40]. Despite these good intentions, our review of real school closures suggests parents are less likely to achieve this, even when schools are closed for much shorter periods of time. Regardless of the conviction with which people answer questions about their likely future actions, much caution is needed in using such data to assume likely behaviours or make decisions about social distancing measures. The duration of planned closure of schools is likely to be important here too; short closures of up to a couple of weeks may be manageable by parents as seen in the studies reviewed but longer closures required for curtailing pandemic waves of the order of months may provide more challenge to them.

Further research is needed to identify how best to ensure that children are incentivised to stay at home during a school closure. The relatively sparse research conducted to date, limited by the real-world occurrence of school closures and the feasibility of conducting rapid research when these do occur, do not allow us to provide a ready answer to this question, but improved communication with both parents and children is likely to be required.

In terms of limitations for this review, the generalisability of the individual studies we identified is unclear. Notably, much may depend on the cultural context, perceptions of the illness in question, length of the closure, socioeconomic status of the families that are affected and information or instructions that are given to them by public health authorities. With relatively few studies in this field, it is difficult to disentangle these effects. The majority of studies examined school closures because of the 2009 H1N1 pandemic and behaviours during this period may not necessarily reflect behaviours during closures for other reasons or even other infectious diseases. Additionally, several studies looked at school closures because of influenza-like illnesses, which may be considered to be mild and not too dangerous in children [41]. Behaviour during closures for this reason may be different to behaviour during closures for diseases perceived as more severe. It must also be noted that the majority of included studies were from the US, perhaps because of our decision to limit the review to English or Italian papers, and thus may not be generalisable to other cultures or countries. Future reviews should consider including papers published in other languages. While we extracted the duration of school closure from studies included in the review (Table), we did not formally analyse whether the length of school closure was associated with children’s activities and contacts made outside the home. The closures we identified lasted for less than 2 weeks, limiting our ability to draw conclusions on this. However, we note that practical issues, including difficulties with childcare and economic impact, were identified by several studies. It seems plausible that longer closures would increase these difficulties. Also, while ideally this review would have calculated a mean reduction in contacts based on all studies or an overall percentage of children who left the home across all studies, this kind of calculation was not possible because of the differences in the way studies measured contacts, the time over which they were measured and the different ways of reporting this information.

No standardised quality appraisal of the studies included in this review was carried out because of the rapid nature of this review, which is common for reviews which need to be carried out urgently in order to guide health policy decisions [42]. However, there were some notable limitations to the literature reviewed. Most were convenience samples, often with low response rates, so may not be representative of all households in the wider community [21,28]. It is likely that particularly vulnerable households would experience greater difficulties and would not have prioritised participating in research studies. Because of this, such groups may not be well-represented in the data. Other limitations included different data collection time points, e.g. collecting data for some participants a week after the closure and others 3 months later [28]; comparing fully-closed schools with partially-closed schools, e.g. schools where only some classes were told to remain at home and extra-curricular activities remained open [20]; and potential under-estimation of social contacts because of only asking about specific planned activities and not incidental activities [22].

Current models frequently use planned school closures, e.g. weekends and school holidays, as a proxy for enforced models [43]. Indeed, planned school holidays may be a fair proxy for short-term closures for some parents but we cannot be sure that this can be extrapolated to longer-term closures, e.g. schools potentially closed for months. Human behaviour is complex and understanding how people respond to an evolving and urgent policy is essential. Basing policy on historical patterns may give false confidence in results and not capture uncertainty adequately. Recent reviews of the incorporation of human behaviour into infectious disease models have advocated the use of appropriate, detailed, real-world behavioural data within infectious disease modelling [44,45]. We hope that our identification of real-world data concerning social contact and mixing behaviour during unexpected school closures will help improve existing models and promote rigorous quantitative research in this area.

## Supplementary Data

320000 Supplementary Material
