# Supplementary material for: The impact of unplanned school closure on children’s social contact: rapid evidence review
Source: Euro Surveill. 2020 Apr 2;25(13):2000188. doi: 10.2807/1560-7917.ES.2020.25.13.2000188 (PMC7140596; doi:10.2807/1560-7917.ES.2020.25.13.2000188)
Supplement: Supplementary Material [file 20-00188_BROOKS_Supplement.pdf]

Supplementary Material

This supplementary material is hosted by *Eurosurveillance* as supporting information alongside the article 'The impact of unplanned school closure on children's social contact: Rapid evidence review', on behalf of the authors, who remain responsible for the accuracy and appropriateness of the content. The same standards for ethics, copyright, attributions and permissions as for the article apply. Supplements are not edited by *Eurosurveillance* and the journal is not responsible for the maintenance of any links or email addresses provided therein.

Supplement S1: Full details of activities outside the home and contacts with non-household members during school closures.

Study: Author (year), place	Participants	Activities outside the home and contacts with non-household members
Basurto-Davila et al. (2013), Argentina [28]	226 households; Children from 3 schools closed for 2 weeks due to H1N1 (School A – children aged 6-12; School B – children aged 13-15; School C – children aged 6-15. Schools A and B were in the city of	67% of children visited public places at least once; 45% left the house several times.<br>Approximate percentages taking part in out-of-home activities (Ushuaia region / Jujuy region respectively):<br>~45% / ~35% visited the supermarket;<br>~20% / ~40% visited plazas / recreation areas;<br>~28% / ~30% visited shopping malls;<br>~28% / 22% attended indoor friend gatherings;<br>~5% / ~30% attended religious events;

	Ushuaia; School C was in Jujuy)	~10% / ~28% used public transport;<br>~20% / ~3% visited movie theaters;<br>~10% / ~12% attended indoor sports practice;<br>~15% / ~8% visited restaurants;<br>~3% / ~2% participated in group activities.<br><br>Childcare arrangements (Ushuaia region / Jujuy region respectively):<br>82% / 88% were left with a relative or family friend;<br>13% / 5% were with a hired nanny;<br>3% / 4% had 'other special arrangements';<br>2% / 1% of children were left alone.
Borse et al. (2011), USA [25]	554 households; Median age of children: 8 years; Schools closed for 5-7 days due to H1N1	30% of students visited at least one locale outside their homes.<br>For children without an influenza-like illness:<br>~20% visited a playground or park;<br>~5% went to a grocery store / mall / ran errands;<br>~3% visited family;<br>~3% visited library,<br>~3% went to a restaurant;<br>~3% attended a religious function or service;<br>~2% attended a sports practice or game;

		~2% went to a movie theatre; and friend's house.<br>~1% went to a friend's house;<br>~1% visited a doctor.<br>Children who were sick with an influenza-like illness before or during the closure were only slightly less likely to visit a playground or park; their second most common venue was a doctor visit which they were significantly more likely to visit than a non-sick child; and they were slightly more likely to visit family than non-sick children.
Braunack-Mayer et al. (2013), Australia [14]	4 school principals, 25 staff, 14 parents, 13 students;<br>Students ranged in age from 12-17;<br>Schools either partially or fully closed due to H1N1 (length of closure unclear)	Qualitative study indicating most people adhered to advised quarantine, but in the absence of clear instructions, many invented their own rules.<br><br>Some parents quarantined their children to avoid being seen as irresponsible. However, many were home alone and so it was unclear whether children complied with this. Others reported seeing the closure as ineffective and did not quarantine their children. One student reported meeting friends regularly even though his parents believed he was at home.

Chen et al. (2011), Taiwan [31]	232 households;<br>School for children aged 5-12;<br>School closed for 7 days due to H1N1	13% went to public places or gatherings at least once:<br>12% visited relatives;<br>5% went to parents' workplace.<br><br>60% were cared for by parents, 35% by other relatives, 4% by others, 1% stayed home alone. (Compared to pre-closure: 39% were cared for by parents after school, and 29% were in afterschool childcare.)
Effler et al. (2010), Australia [20]	233 households;<br>Median age of children: 11 years (range 5-13);<br>3 schools closed due to H1N1;<br>School A closed entirely 'for the coming week';<br>Schools B and C cancelled classes only for grade 5 and grades 5-7 respectively	74% participated in activities outside the home on at least one occasion, reporting a total of 860 out-of-home activities with an average of 3.7 out-of-home activities for each student.<br>Activities reported (n during the week / n at weekend):<br>Park or beach (144 / 52);<br>Sporting event (86 / 128);<br>Grocery store (68 / 35);<br>Shopping mall (63 / 28);<br>Unspecified event or activity (58 / 17);<br>Party (28 / 37);<br>Music or art lesson (19 / 8);<br>Restaurant (13 / 14);<br>Cinema (16 / 5);

		Religious service (1 / 16);<br>Sleepover (10 / 6);<br>Tutoring lesson (6 / 2).<br><br>19% of 202 asymptomatic students were cared for in a setting with children other than their siblings; 6% of 31 ill students were cared for with children other than their siblings. 10% cared for themselves at home for at least a portion of the closure period.
Epson et al. (2015), USA [21]	35 households, representing 67 students;<br>1 elementary school and 1 junior and senior high school that are housed in the same building complex;<br>Schools closed between 29.01.13 and 05.02.13 due to influenza-like illness	58% visited at least one outside venue:<br>58% went to grocery stores;<br>33% went to restaurants;<br>25% went to sports practices;<br>25% went to religious services;<br>25% visited friends' houses.<br><br>Childcare involving contact with non-household members:<br>9% were looked after by an adult who lives outside the household;<br>6% went to work with their parents;<br>3% were in a childcare program.<br>9% were left home alone.

Gift et al. (2010), USA [24]	214 households, representing 269 children under 18; Elementary school closed for 1 week due to H1N1	69% visited at least one other location.<br>The most common activities outside of the home were: ‘other’; shopping, sports activities, restaurants, visiting family, visiting healthcare provider, visiting friends, going to a movie or concert, visiting the library, attending a religious venue, going to a party, and going to a drive-through restaurant.<br><br>Home was the primary location for 77%; the next most common location was another family member's home, followed by non-family member's home, parents’ workplace, vacation, daycare, and ‘other’.
Jackson et al. (2011), UK [17]	107 students (only 46 reported how many times they visited public places during closures);<br>Children aged 11-15;<br>School closed for 1 week, reopened for 2 days, then closed	98% visited more than one place.<br>78% visited shops (compared to 94% when school was open);<br>59% visited a park or playing field (81% when school was open);<br>46% visited places of worship (66% when school was open);<br>43% used public transport (58% when school was open);<br>37% visited cafes (50% when school was open);

	for another week, due to H1N1	28% visited leisure centres (42% when school was open);<br>26% went to parties (22% when school was open);<br>17% visited movie theatres (17% when school was open).<br><br>Fewer students visited shops, places of worship, parks and playing fields at least once per week when school was closed than when open; for other places frequency of visits did not differ.<br><br>Among caregivers for whom information was available, 125/182 (69%) would have seen the student on a typical school day.<br><br>73 students provided their typical number of contacts per day during closure and 35 also provided information for a typical school day. Mean totals of reported contacts were 70.3 and 24.8 during typical school days and closure respectively.

Johnson et al. (2008), USA [23]	220 households, representing 355 children;<br>Median age of children: 12 (range 5-19);<br>Schools closed between 01.11.06 and 12.11.06 due to influenza virus B	89% visited at least one public location despite recommendations to avoid large gatherings and 47% travelled outside of the county.<br>44% visited grocery stores;<br>35% visited fast food restaurants;<br>34% visited church services;<br>19% visited malls;<br>15% visited parties or sleepovers.<br><br>10% had to make special childcare arrangements including grandparents or other relatives or other adults, taking the child to work, having older siblings watch them or using childcare programs.<br>3% had to have their child spend more than one night outside the household for childcare purposes.
Litvinova et al. (2019), Russia [18]	450 participants including students and their household members;<br>2 schools – School A for children aged 6-17 and School B	When school closure policy was in place, there was a significant reduction in the number of contacts made by students (14.2 contacts per day when open vs. 6.5 when school was closed). School-closure policies induced students to significantly reduce their number of contacts with individuals aged under 18 (75% reduction) and 19-59 year-olds (20%

	for children aged 6-15; Gradual school closure policies performed every year to mitigate spread of seasonal influenza; Schools closed for 7 days	reduction), while simultaneously increasing contacts with individuals aged 60 or over (52% increase), although thee absolute value remained low (i.e., less than one contact per day).
McVernon et al. (2011), Australia [29]	314 households; 33 schools with children under 18; Schools with confirmed cases of H1N1 in multiple classes were entirely closed for 7 days while schools with confirmed cases in only one class were instructed to close only that class	43 households reported that a child spent at least one day outside the family home, and mixing with other children occurred on almost half of these occasions (48.8%), whether or not there was an influenza case in the family. Contact with children who were not immediate family members was far less likely during days spent at home. No child visited a household in which another child was ill, compared with reported child visitors in 15.9% of 226 homes without a case.<br><br>Compared to children in households that complied with recommendations to stay at home, children in households that did not

		comply with the recommendations were more likely to have been cared for during the quarantine period by an adult from outside the home (28.3% compared with 4.0% for compliant households; $p < 0.001$), thus also contravening the quarantine recommendation not to mix with adults from outside the household. This distinction was especially marked for households in which there was a confirmed case of influenza, where the difference was 44.4% of children receiving outside care in non-compliant households compared with 2.4% of those that were compliant.
Miller et al. (2010), USA [19]	63 parents of 176 lower school students (grades 5-8);<br>188 upper school students (grades 9-12);<br>Week-long closure due to H1N1	Mean number of days spent on activities (upper school):<br>3.42 any other outdoor activity;<br>2.44 eating at restaurants;<br>1.89 using public transport;<br>1.48 hosting a friend;<br>1.47 shopping;<br>1.47 any other indoor activity;<br>0.44 working at a job.

		Average number of friends seen per day (upper school): 2.53 Wednesday, 2.06 Thursday, 2.59 Friday, 2.40 Saturday, 1.23 Sunday, 1.02 Monday, 1.05 Tuesday.<br><br>Proportion of caregivers (upper school): 0.62 parent, 0.24 sibling, 0.07 grandparent, 0.07 other, 0.06 nanny or babysitter, 0.07 friend's caretaker, 0.11 other, 0.88 self.<br><br>Mean number of days spent on activities (lower school):<br>2.77 any other outdoor activity;<br>1.34 eating at restaurants;<br>1.12 any other indoor activity;<br>1.05 shopping;<br>0.73 visiting a friend;<br>0.55 hosting a friend;<br>0.10 using public transport.<br><br>Average number of friends seen per day (lower school): 0.30 Wednesday, 0.52 Thursday, 0.84 Friday, 0.83 Saturday, 1.17 Sunday, 0.74 Monday, 0.68 Tuesday.

		Proportion of caregivers (lower school):<br>0.85 parent, 0.30 sibling, 0.09 grandparent, 0.15 other family, 0.27 nanny or babysitter, 0.03 friend's caretaker, 0.06 other, 0.76 self.
Mizumoto et al. (2013), Japan [26]	882 households; 25.2% in kindergarten, 24.8% in primary school, 25.1% in junior high school and 24.9% in high school, age range 4-18;<br>‘School closure or class suspension at least once’ due to H1N1	20.5% left the home for non-essential reasons. Of the 351 students who left the home:<br>40.9% went shopping at a supermarket or convenience store;<br>19.3% went shopping at a department store or large shopping centre;<br>18.7% attended extra classes, prep school, or English school;<br>9.5% visited an outdoor playground or pool;<br>8.7% participated in a sports activity;<br>8.2% ate at restaurants;<br>2.0% participated in a concert, drawing, or other artistic activities;<br>1.0% had an overnight stay away from home;<br>0.5% participated in religious activities;<br>0.3% participated in an outdoor group activity;<br>0.3% visited the cinema;<br>0.1% went to a party;<br>11.2% took part in other activities outside the house.

		28.5% children looked after themselves;<br>64.3% were looked after by another household member; 7.3% required a special arrangement such as parental absence from work.
Russell et al. (2016), USA [27]	99 households, representing 197 children;<br>Students in pre-kindergarten up to 12th grade;<br>School closed for 4 days due to influenza-like illness	77% of children went outside the home or visited with a non-household member, participating in a mean of two activities (IQR 1-4):<br>54% visited a mall or department store;<br>51% visited a grocery store;<br>32% attended religious services;<br>32% visited family;<br>24% went to restaurants or sports activities.<br><br>Childcare involving contact with non-household members:<br>20% were looked after by a non-household adult;<br>1% were in a childcare program.
Steelfisher et al. (2010), USA [32]	523 parents;<br>Ages not reported;<br>Childcare centres and schools closed due to H1N1 - 10% were closed for 1	56% reported their child participated in at least one activity involving people outside the household:<br>30% visited other children's houses;<br>30% went grocery shopping;<br>23% went to fast food restaurants;

	day, 19% for 2 days, 29% for 3 days, 15% for 4 days, 17% for 5 days, 9% for more than 5, 2% didn't know	17% went to public events such as movies, sporting events or concerts;<br>15% visited large shopping areas or malls;<br>6% attended social events such as parties or dances.<br><br>81% were cared for by an adult in the household, 20% by a family member outside the household, 1% by a friend/neighbour, 3% by a professional care provider, and 10% stayed home alone.
Timperio et al. (2009), USA [30]	262 households representing 480 children;<br>Ages not reported;<br>Two schools closed due to seasonal influenza; one closed for 3 days and the other closed for 4 days	43.3% visited strip malls or Wal-Mart (the largest store in the area);<br>42.9% visited family;<br>38.7% went grocery shopping;<br>32.6% ate at restaurants;<br>30.3% either visited friends' houses or had friends visiting their house;<br>29.1% attended religious services;<br>23.8% took part in sports activities;<br>17.6% went to public gatherings such as concerts, movies or festivals;<br>8.4% went to a part time job.

Tsai et al. (2017), USA [33]	208 households representing 423 students; Children aged under 18; School closed for 8 days due to influenza	3% of children were put into a childcare program; 1% went to work with their parents. 1% were left at home without supervision and another 15% were old enough to care for themselves.
van Gemert et al. (2018), Australia [22]	99 students with laboratory confirmed pH1N1; Age 6-17; 49% were aged 14-15; 33% were aged 16-17; 9% were aged 12-13; 5% were aged 10-11; 4% were aged 6-7; Seven schools closed for a range of 3-9 days (not including weekends) due to H1N1	26% (21/81) who reported usually taking part in extra-curricular activities (not sports or religious activities) continued to take part in extra-curricular activities; 23% (14/62) continued to participate in sports activities; 20% (2/10) continued to take university classes; 11% (2/18) continued to attend part-time work; 5% (1/20) continued to take part in religious activities.

Zheteyeva et al. (2017), USA [34]	2,229 households representing 4,247 students; Kindergarten – Grade 12; Schools closed for 4 days in preparation for Hurricane Isaac	5.3% of children went to work with their parents; 2.6% of children were put in a childcare program. 11.6% were old enough to care for themselves, while 2.5% were left at home without supervision.
